# Evaluation of the meibomian glands using the tear interferometer wearing orthokeratology lenses

**DOI:** 10.1186/s12886-022-02365-3

**Published:** 2022-03-24

**Authors:** Jiyoung Lee, Gyudeok Hwang, Minji Ha, Hyun-Seung Kim, Kyungdo Han, Kyung-Sun Na

**Affiliations:** 1 Department of Ophthalmology, College of Medicine, The Catholic University of Korea, Seoul, 06591 Republic of Korea; 2 Department of Statistics and Actuarial Science, Soongsil University, Seoul, 06978 Republic of Korea

**Keywords:** Orthokeratology, Meibomian gland, Pediatric, Dry eye, Tear film, Lipid layer thickness, Blinking

## Abstract

**Background:**

To investigate the impact of orthokeratology wear on meibomian glands in Korean pediatric population using the tear interferometer.

**Methods:**

Fifty-three orthokeratology wearers and 79 non-lens wearers were evaluated using the LipiView® II ocular surface interferometer which shows incomplete blink rate, average lipid layer thickness, and meiboscores.

**Results:**

No significant differences in the incomplete blink rate and meiboscores for upper eyelids, but the lipid layer thickness and meiboscores for lower eyelids were significantly higher in the Ortho-K group than in the control group (*p* = 0.024 and 0.007, respectively). Correlation analysis showed no significant correlation between the duration of orthokeratology wear and the parameters measured by LipiView® (*p* > 0.05 for all). Among subgroups based on average duration of lens wear, the longer duration (≥ 24 months) subgroup showed higher meiboscores of lower eyelids (*p* = 0.011), but no other significant differences.

**Conclusions:**

Ortho-K wearers showed no significant differences in the incomplete blink rate and meiboscores of upper eyelids, but they were associated with increased LLT and higher meiboscores of lower eyelids. Thorough examination and close monitoring of orthokeratology wearers is necessary. Prospective and observational studies are needed to further elucidate the relationship between Orthokeratology and meibomian glands.

**Supplementary Information:**

The online version contains supplementary material available at 10.1186/s12886-022-02365-3.

## Background

Orthokeratology (Ortho-K) is a treatment for myopia that uses reverse geometry gas-permeable contact lenses (CLs) to temporarily reshape the corneal contour. The lenses are worn overnight to flatten the central cornea and reduce myopia, and their effectiveness has been well documented in previous studies [1–3]. However, there are concerns about the pathologic changes to the ocular surface and meibomian glands (MGs) associated with overnight Ortho-K lens wear [4]. 

MGs are modified, holocrine, sebaceous glands that are embedded in the tarsal plate [5] and secrete meibum into the tears to prevent excessive evaporation of tear fluid. Meibomian gland dysfunction (MGD) is a chronic, diffuse abnormality of the MGs, commonly characterized by terminal duct obstruction and/or changes in the glandular secretion [6] and it has attracted attention as a leading cause of dry eye disease (DED) [7, 8]. CL wear is one of the potential causes of alteration in MGs, and may result in MGD [9]. Moreover, CL wear can decrease corneal sensitivity [10, 11], affect blink frequency [12], and induce an allergic reaction from MG distortion [13].

The LipiView® II Ocular Surface Interferometer (Tear Science Inc. Morrisville, NC, USA) enables quantitative measurement of the average of lipid layer thickness (LLT) by assessing tear film interference, visualizing the MG structure and morphologic changes, and assessing blinking patterns of individuals [14, 15]. We compared the structure (infrared meibography) and function (LLT) of MGs, and the blinking pattern in the Korean pediatric population between those wearing Ortho-K lenses and normal controls. Additionally, we evaluated the correlation between the duration of Ortho-K wear, and the meiboscore, LLT, and incomplete blink rate. The aim of this study was to investigate the impact of orthokeratology wear on MGs in the Korean pediatric population using the tear interferometer.

## Methods

### Subjects

This study is a retrospective review of clinical records conducted at Yeouido St. Mary’s Hospital, Seoul, South Korea. This study adhered to the tenets of the Declaration of Helsinki and approval for retrospective review of clinical records was obtained from the Institutional Review Board (IRB) of the Catholic University of Korea (SC21RISI0001, Seoul, South Korea). Written informed consent obtained from parents/legal guardians for study participation was waived by IRB of the Catholic University of Korea (SC21RISI0001, Seoul, South Korea) because of the retrospective nature of the study.

The study included pediatric population aged 7 to 17 years who visited the Yeouido St. Mary’s Hospital between September 2018 and December 2020. Subjects with a history of intraocular or eyelid surgery, ocular infectious (viral or bacterial) or inflammatory diseases, as well as those diagnosed with dermatologic diseases including pediatric rosacea or allergic diseases and/or taking medications such as antihistamines, were excluded. Patients who showed any signs or symptoms of DED or MGD were also excluded.

The control group comprised of subjects who visited the clinic for the prescription of Ortho-K and they did not have a history of lens wear, irrespective of whether they wore glasses. Subjects in the Ortho-K group had been using the lenses for at least 4 months. The final cohort comprised 79 patients of control group and fifty-three patients of Ortho-K group. Right eye of each patients were used in the analysis.

### Lenses

We prescribed rigid gas-permeable CLs with a reverse geometry design (Lucid LK; Lucid Korea, Seoul, South Korea) to all subjects and checked the lens-fitting according to the manufacturer’s guidelines. All patients wore the lenses for at least seven continuous hours while sleeping and immediately removed the lenses on awakening. The treatment policy at our lens clinic is that we neither prescribed the lens to the candidate subjects nor discontinued its use in the Ortho-K group participants with symptoms of DED or MGD [16–19]. Thorough opthalmic examination were performed at any time of visits.

### Data collection

Information of the LipiView® II Ocular Surface Interferometer (Tear Science Inc. Morrisville, NC, USA) was used for the evaluation of the lipid layer thickness (μm), and the percentage of incomplete blinks (incomplete blink rate).

Both the upper and lower eyelids were everted and MGs were observed using a noncontact meibography system. Partial or complete loss was scored according to the meiboscores for the upper and lower eyelids eyelids from the LipiView images as follows [20]: 0, no gland dropout; 1, dropout with less than 25% of the loss; 2, dropout with 26% to 50% loss; 3, dropout with 51% to 75% loss; and 4, dropout with more than 75% loss.

### Statistical analysis

We used propensity score (PS) weighting to compare between the variables [21, 22]. We assessed the PS between the control and Ortho-K groups, using ordinal logistic regression based on the baseline covariates, including age, BCVA, axial length, and astigmatism. In addition, we used the inverse probability weighting (IPTW) to balance the baseline covariates among the two groups during time-to-event analyses using stabilized weights calculated from the PS [23]. The data were weighted according to this technique. Comparisons of demographic characteristics (age, gender, BCVA, axial length, and astigmatism) and parameters measured by the LipiView (incomplete blink rate and average lipid layer thickness of lower and upper eyelids) between the two groups were analyzed using unpaired t-tests. As meiboscores was considered ordinal variables, it was analyzed by Cochran-Mantel–Haenszel Chi-square test with trend *p*-value. Pearson’s correlation analysis was used to determine the relationship of parameters measured by the LipiView with the duration of Ortho-K lens wear. A *p*-value of < 0.05 was considered statistically significant.

## Results

### Demographic characteristics

**Fig. 1:**
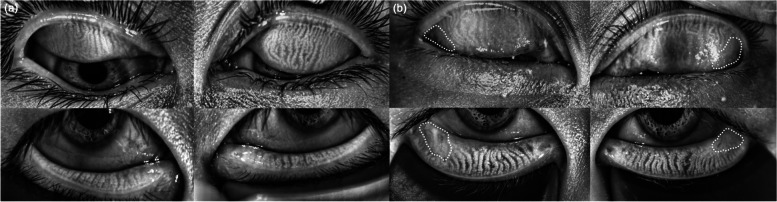
Representative cases involving children examined by the LipiView® II Ocular Surface Interferometer without (candidate group) and with orthokeratology lens wear (Ortho-K group). **a** Images for a 14-year old girl from the control group. The average lipid layer thicknesses in the right and left eyes of the girl were 92 and 97 nm, respectively. In addition, she did not show any signs of MGs loss in the upper and lower eyelids. **b** Images for a 10-year old boy from the Ortho-K group. He showed average lipid layer thicknesses of 59 and 55 nm in the right and left eyes, respectively, with corresponding incomplete blinking rates of 100% and 25%. A 25% MGs loss was noted in the distal portion of the upper and lower eyelids (white outlined contour)

In total, 79 eyes were enrolled in the control group (aged 7–14 years) and fifty-three eyes in the Ortho-K group (aged 8–17 years) (Fig. 1). Detailed demographic characteristics of patients in both groups are presented in Table 1.

**Table 1:** Demographic characteristics without (Control group) and with (Ortho-K group) orthokeratology treatment in Korean children

	**Control group** **(*n* = 79)**	**Ortho-K group** **(*n* = 53)**	***p*-value**
Age (years)	9.92 ± 1.94	9.97 ± 2.05	0.899
Gender, female (%)	70.26	66.47	0.644
BCVA (logMAR)	0.06 ± 0.1	0.07 ± 0.07	0.897
Axial length (mm)	24.78 ± 1.01	24.67 ± 0.8	0.502
Astigmatism (diopter)	1.42 ± 0.88	1.39 ± 0.71	0.841

*Ortho-K* Orthokeratology, *BCVA* distance best-corrected visual acuity, *logMAR* logarithm of the minimum angle of resolution

### Comparison analysis of meibomian glands

Table 2 and supplementary Table S1 presents the weighted values for the parameters measured by the LipiView for both groups. There were no significant differences in the incomplete blink rate and meiboscores of upper eyelids between the two groups. However, the LLT and meiboscores of lower eyelids were significantly higher in the Ortho-K group than in the control group (*p*-value = 0.024 and 0.007, respectively).

**Table 2:** Parameters measured by the LipiView® II Ocular Surface Interferometer without (Control group) and with (Ortho-K group) orthokeratology treatment in Korean children

	**Control group** **(*n* = 79)**	**Ortho-K group** **(*n* = 53)**	***p*-value**
Incomplete blink rate (%)	55.81 ± 31.47	53.54 ± 36.37	0.702
Average lipid layer thickness (μm)	67.98 ± 21.46	76.7 ± 21.93	0.024^*^
Meiboscores, lower eyelids (%)			0.007^*^
0	29.7	14.79	
1	58.27	60.4	
2	12.04	19.74	
3	0	5.06	
Meiboscores, upper eyelids (%)			0.115
0	61.16	45.23	
1	32.86	47.49	
2	5.98	7.29	
3	-	-	

^*^Statistically significant; Ortho-K, Orthokeratology

### Correlation analysis

The average duration of lens wear in the Ortho-K group was 24 months, with a minimum of 4 months and a maximum of 48 months. The findings of correlation analyses are shown in Table 3; there was no significant association of the duration of Ortho-K lens wear with the incomplete blink rate (*R*^2^ = 0.009; *p* = 0.499), average lipid layer thickness (*R*^2^ = 0.04; *p* = 0.150), and meiboscores for the upper and lower eyelids (*p* > 0.05). We subsequently divided the patients in the Ortho-K group according to the average duration of lens wear: long (≥ 24 months) and short (< 24 months). The detailed and weighted demographic characteristics of patients in both the groups are summarized in Table 4. Table 5 showed significantly higher lower-eyelid meiboscores in the longer duration subgroup (*p* = 0.011), but other parameters did not show significant differences between the longer duration (*n* = 26) and shorter duration (*n* = 27) groups.

**Table 3:** Correlation of the duration of orthokeratology (Ortho-K) lens wear with the incomplete blink rate, lipid layer thickness, and meiboscores in Korean children

	**Pearson’s correlation**	***p*-value**
Incomplete blink rate (%)	0.0949	0.499
Average lipid layer thickness (μm)	0.2004	0.150
Meiboscores, lower eyelids (0–3)	0.1685	0.228
Meiboscores, upper eyelids (0–3)	-0.2131	0.126

**Table 4:** Demographic characteristics according to the duration of orthokeratology (Ortho-K) lens wear in Korean children

	**Short duration**^a^ **(*n* = 27)**	**Long duration**^a^ **(*n* = 26)**	***p*-value**
Age (years)	12.05 ± 3.51	11.15 ± 1.93	0.249
Gender, female (%)	82.33	84.81	0.805
BCVA (logMAR)	0.06 ± 0.07	0.06 ± 0.05	0.979
Axial length (mm)	25.09 ± 0.92	24.98 ± 0.67	0.593
Astigmatism (diopter)	1.34 ± 0.61	1.35 ± 0.82	0.964

*Ortho-K* Orthokeratology, *BCVA* distance best-corrected visual acuity, *logMAR* logarithm of the minimum angle of resolution

^a^Short duration(< 24 months)/ Long duration(≥ 24 months)

**Table 5:** Incomplete blink rate, lipid layer thickness, and meiboscores according to the duration of orthokeratology (Ortho-K) lens wear in Korean children

	**Short duration**^a^ **(*n* = 27)**	**Long duration**^a^ **(*n* = 26)**	***p*-value**
Incomplete blink rate (%)	57.44 ± 41.99	54.26 ± 32.24	0.755
Average lipid layer thickness (μm)	79.0 ± 26.09	83.54 ± 19.19	0.468
Meiboscores, lower eyelids (%)			0.011^*^
0	34.68	9.65	
1	51.23	53.44	
2	12.04	26.63	
3	2.05	10.27	
Meiboscores, upper eyelids (%)			0.421
0	41.18	57.46	
1	48.03	30.38	
2	10.8	12.16	
3	-	-	

^a^Short duration(< 24 months)/ Long duration(≥ 24 months)

^*^Statistically significant

## Discussion

The prevalence of myopia in adolescents is increasing worldwide [24] and Ortho-K is currently accepted as a safe and effective measure for myopia correction in the pediatric population [25, 26]. So far, although many studies have been conducted to investigate the effects of CL wear on MG structure and function, their findings have been controversial. Arita et al. [27] found that CL wear is associated with a decrease in the number of functional MGs while Machalinska et al. and Pucker et al. [28–31] concluded that CL wear does not appear to influence MGs. Alghamdi et al. [32] found alterations in MG morphology and function but it during the first two years of CL wear. These controversies were likely caused by the differences in the inclusion and exclusion criteria among them. Moreover, little research has been conducted on the effects of overnight wear of reverse geometry CLs in patients under 18 years of age.

In the current study, the LLT was 67.98 nm in the control group and 76.7 nm in the Ortho-K group; although these values are within the normal range, they are higher than the average value reported by Zhao et al. [33]. Interestingly, the average LLT was significantly higher in the Ortho-K group than in the control group, despite fewer MGs in the lower eyelids. This might result from true hypersecretion of the MGs, or damming back of secretions in the presence of mild obstruction [6]. Furthermore, a trend of higher lipid secretion scores was observed in those wearing Ortho-K lenses than at the baseline in teenagers with myopia [34]. Inflammatory mediators may promote subclinical inflammatory changes in the MGs and lead to qualitative changes in the composition of meibum [35]: moreover, CL wear is a risk factors for ocular surface inflammation [36]. A compensatory mechanism can also explain the result of the present study. A previous study suggested that obstruction of the lower eyelid orifice might result in increased lipid secretion in the upper eyelid for the maintenance of homeostasis [37]. CL wear may induce mild obstruction of MG orifices due to the aggregation of desquamated epithelial cells [9, 38].

According to Arita et al. [27], the meiboscores for CL wearers were significantly higher for the upper eyelid, suggesting that the upper eyelid may experience CL-mediated irritation of MGs. However, we found higher lower-eyelid meiboscores in the Ortho-K group than in control group which is consistent with a previous study showing MG distortion on the distal aspect of the lower eyelids [4]. One possibility is that these patients may have had subclinical allergic conjunctivitis and distorted MGs before lens wear and Ortho-K treatment merely aggravated the pre-existing condition, causing further distortion or structural abnormalities in MGs. Wu et al. [39] found that MG loss occurred in both children and adolescents using a non-contact infrared meibography system, despite the lack of abnormalities in ocular surface parameters. These findings suggest that MG dropout may exist without any history of DED or MGD in pediatric patients. In addition, Gupta et al. [40] reported relatively, high levels of atrophic changes in MGs as a normal variation in the pediatric population. Zhao et al. [33] found that only 16 of 266 asymptomatic children had no MG deficiency. Wang et al. [41] reported an average meiboscore of 0.61 for the control group. Although no significant correlation was observed between duration of Ortho-K wear and lower eyelids meiboscores before IPTW (Table 3), the meiboscores of the lower eyelids showed higher values in the long duration of Ortho-K wear subgroup than short duration after IPTW (Table 5). Future studies should focus on prospective and observational designs to assess the changes and correlations between LLT and meiboscores in pediatric population.

This study has several limitations. The retrospective and cross-sectional study design cannot confirm causal relationship between Ortho-K lens use and changes in MG morphology and function. The number of subjects was small, and the observation period was relatively short (maximum 48 months). Long-term follow-up is required to explore the reversibility of the changes and differences between the control group and the Ortho-K group. A more detailed analysis according to wearing period is also needed and future studies should focus on a larger sample size. Some younger subjects may have mild subnormal vision due to poor cooperation, not for the ocular surface abnormality. Visual acuity should be measured more precisely. Failure to account for the longitudinal variations of measurements in the same subjects can be accredited to the parameters being evaluated once; therefore, so prospective studies should analyze the changes over time to strengthen the research. In addition, we used subjective and objective ocular surface parameters and tear dynamics including the Schirmer test I, tear break-up time, and Oxford scale with ‘Pass / Fail grading’ for Ortho-K fitting. However, detailed values of these parameters were not recorded. Moreover, another limitation was that the children with dry eye might display fewer symptom scores than adults [42]. A recent study reported that when applying the Ocular Surface Disease Index to children, there was lower repeatability and it needed more assistance than adults [43]. Consequently, new symptom questionnaires and diagnostic criteria that specifically reflect the characteristics of pediatric patients and are easy for them to understand should be developed. Use of the LipiView® device could facilitate data collection for children with low compliance.

Although the quality of the meibum was not evaluated in this study, future studies should examine it with the values of meiboscores to evaluate the mechanical effects of orthokeratology lenses in MGs. The LipiView® device was used to assess the “normal” subjects who visited the clinic for the prescription of Ortho-K lenses. This may have introduced a selection bias. In addition, considering the relatively young age of the subjects, they might have shown poor cooperation resulting in an unnatural blinking pattern. The use of infrared video recordings collected by the Keratograph 5 M (Oculus, Wetzlar, Germany) might facilitate observation of a more natural blinking pattern [44]. Furthermore, video recordings [45] could allow the rapid and noninvasive observation of MGs, thus revealing a more detailed relationship between the incomplete blinking rate and Ortho-K lens wear. Future studies should thoroughly examine the detailed location and pattern of findings in meibography with the simultaneous use of multiple recording systems. Finally, a corneal and external eye specialist graded the images to minimize bias. However, intra-grader and inter-grader variability should be considered.

## Conclusion

In conclusion, Ortho-K wearers showed no significant differences in the incomplete blink rate and meiboscores of upper eyelids, but they were associated with increased LLT and higher meiboscores of lower eyelids. Future prospective and observational studies are needed to evaluate the relationship between Ortho-K wear and the changes in MGs morphology and function, including ocular surface parameters and tear dynamics. Also, thorough examination of the ocular surface and MGs is warranted before prescribing Ortho-K because there were MGs dropout without any history of DED or MGD. Regular follow-up assessment and close monitoring of MGs are also needed to evaluate the changes in the ocular surface in Ortho-K wearers.

## Supplementary Information

**Additional file 1:** **Table S1. **The median and range values for parameters measured by the LipiView® II Ocular Surface Interferometer without (Control group) and with (Ortho-K group) orthokeratologytreatment in Korean children.

## Data Availability

The datasets used and/or analyzed during the current study are available from the corresponding author on reasonable request.

## Abbreviations

CL: Contact Lens
DED: Dry Eye Disease
IPTW: Inverse Probability weighting
LLT: Lipid Layer Thickness
MGD: Meibomian Gland Dysfunction
MGs: Meibomian Glands
Ortho-K: Orthokeratology
PS: Propensity Score
