# Supplementary material for: Evaluation of the meibomian glands using the tear interferometer wearing orthokeratology lenses
Source: BMC Ophthalmol. 2022 Mar 24;22:133. doi: 10.1186/s12886-022-02365-3 (PMC8951697; doi:10.1186/s12886-022-02365-3)
Supplement: Supplementary file 1 — Additional file 1: Table S1. The median and range values for parameters measured by the LipiView® II Ocular Surface Interferometer without (Control group) and with (Ortho-K group) orthokeratologytreatment in Korean children. [file 12886_2022_2365_MOESM1_ESM.docx]

**Supplementary Table S1. The median and range values for parameters measured by the LipiView® II ocular surface interferometer without (Control group) and with (Ortho-K group) orthokeratology treatment in Korean children**

|  | Control group (n=79) | | Ortho-K group (n=53) | |
| --- | --- | --- | --- | --- |
|  | Median | Range* | Median | Range* |
| Incomplete blink rate (%)  Average lipid layer thickness (μm)  Meiboscores, lower eyelids (0–3)  Meiboscores, upper eyelids (0–3) | 52.94  67  1  0 | 0-100  29-100  0-2  0-2 | 50  86  1  1 | 0-100  28-100  0-3  0-2 |

*Range; maximum to minimum; Ortho-K, Orthokeratology; BCVA, distance best-corrected visual acuity; logMAR, logarithm of the minimum angle of resolution
